# Ischemic Posterior Circulation Stroke: A Review of Anatomy, Clinical Presentations, Diagnosis, and Current Management

**DOI:** 10.3389/fneur.2014.00030

**Published:** 2014-04-07

**Authors:** Amre Nouh, Jessica Remke, Sean Ruland

**Affiliations:** ^1^Department of Neurology, Stritch School of Medicine, Loyola University Chicago, Maywood, IL, USA; ^2^Department of Emergency Medicine, Advocate Christ Medical Center, Oak Lawn, IL, USA

**Keywords:** posterior circulation, stroke, basilar artery, vertebral artery, stroke management

## Abstract

Posterior circulation strokes represent approximately 20% of all ischemic strokes (1, 2). In contrast to the anterior circulation, several differences in presenting symptoms, clinical evaluation, diagnostic testing, and management strategy exist presenting a challenge to the treating physician. This review will discuss the anatomical, etiological, and clinical classification of PC strokes, identify diagnostic pitfalls, and overview current therapeutic regimens.

## Defining the Posterior Circulation

**Figure 1:**
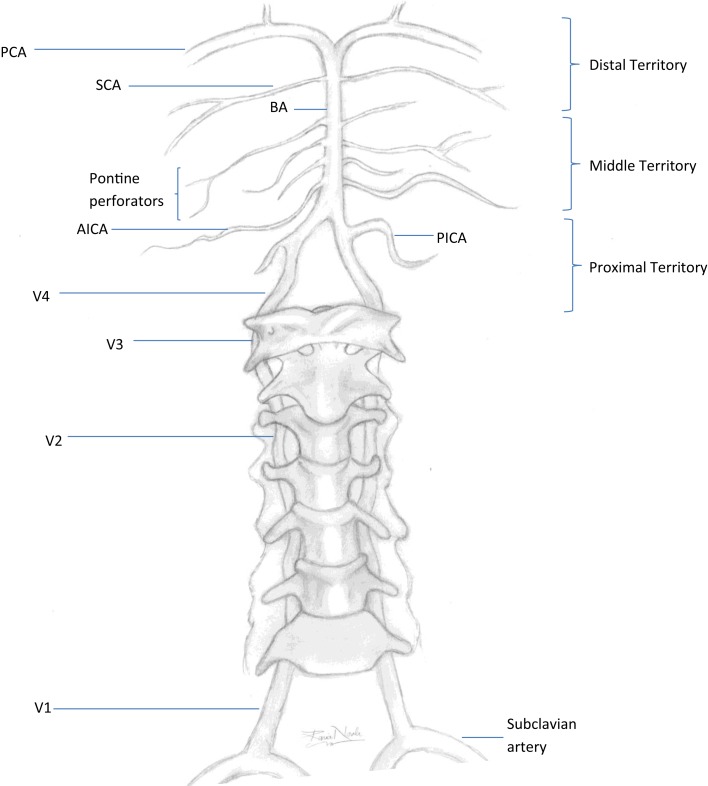
**Vertebrobasilar system**. PCA, posterior cerebral artery; SCA, superior cerebral artery; BA, basilar artery; AICA, anterior inferior cerebellar artery; PICA, posterior inferior cerebellar artery; V1–V4, segments of the vertebral artery. Proximal territory, areas supplied by the intracranial VAs and PICAs up to the VB junction; middle territory, BA and AICAs up to the SCAs; distal territory, rostral BA, SCAs, and PCAs.

A posterior circulation (PC) stroke is classically defined by infarction occurring within the vascular territory supplied by the vertebrobasilar (VB) arterial system. The vertebral arteries (VAs) arise from the right and left subclavian arteries and travel cranially through the transverse foramina of the cervical vertebrae. When reaching the foramen magnum, they pierce the dura mater to start their intracranial course. Both VAs join at the pontomedullary junction forming the basilar artery (BA). The VAs are divided into four segments. V1 is the most proximal segment from the VA origin to point of entry into the initial foramen transversarium, usually at the sixth or seventh cervical vertebral body. The segment occupying the course of the VA from the initial to last transverse foramen is known as V2. The V3 segment begins after the artery exits the foramen transversarium of the axis (C2) and arches behind the atlas (C1) before entering the cranium. The most distal segment representing the intracranial portion of the VA after entering the dura mater is the V4 segment. The BA travels rostral along the ventral medulla and basis pontis until it bifurcates into the right and left posterior cerebral arteries (PCAs) at the pontomesencephalic junction. Segments of the vertebral artery and branches of the vertebrobasilar system are depicted in Figure 1. The topographic classification described by Caplan in the New England Medical Center-posterior circulation registry (NEMC-PCR) (3) divides the intracranial vertebrobasilar system into proximal, middle, and distal territories.

## Anatomical Variants of the Posterior Circulation and Clinical Significance

Anatomical variations of the PC are frequent but commonly asymptomatic. Familiarity with these variants is important as not to mistake them as pathological findings. Although typically discovered incidentally, they are infrequently related to intracranial vascular pathology.

**Figure 2:**
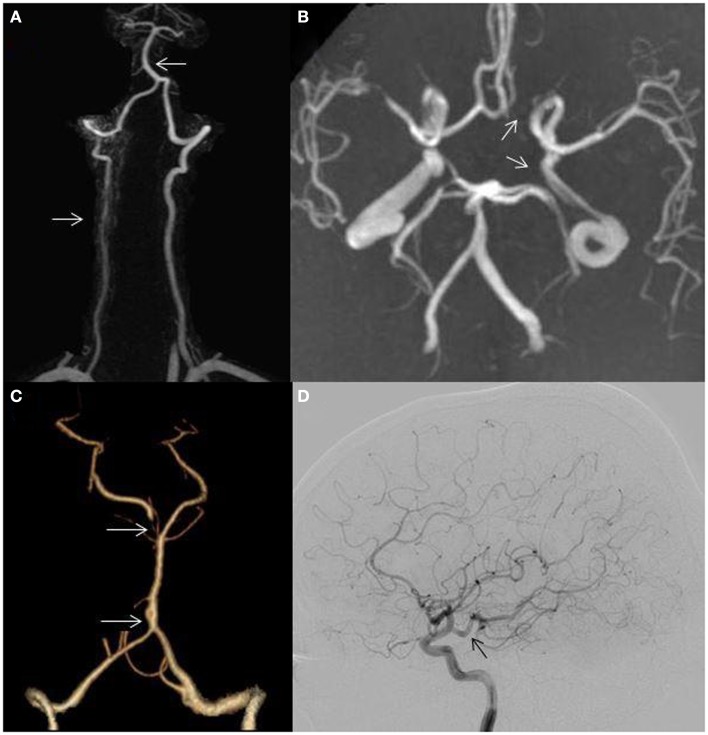
**(A)** Hypoplastic right vertebral artery (bottom arrow); basilar artery displacement opposite to the dominant vertebral artery (top arrow); **(B)** incomplete circle of Willis, absent left posterior communicating artery (bottom arrow), absent left A1 segment (top arrow); **(C)** fenestration of the basilar artery (bottom arrow); hypoplastic right P1 segment (top arrow) and **(D)** posterior cerebral artery arising directly from the internal carotid artery (fetal variant, arrow).

Asymmetric VAs occur in over two-thirds of individuals (4, 5) (Figure 2A) and an incomplete circle of Willis (COW) may be seen in over half (6) (Figure 2B). A persistent trigeminal artery is the most common carotid–vertebrobasilar anastomosis (7). Other intracranial anomalies include fenestration of the vertebrobasilar junction (0.3–0.6%) which may predispose to PC aneurysm formation (8) (Figure 2C), persistent hypoglossal artery, the artery of Percheron, fetal origin of one or both PCAs (fPCA) (Figure 2D), and absence of one or both posterior communicating arteries (PCOMs) (Figure 2B). The labyrinthine artery usually arises from the anterior inferior cerebellar artery (AICA); however this vessel may arise directly from the BA. Pertinent anatomical variants and their clinical significance are highlighted.

### Artery of percheron

*The artery of Percheron* is where a single thalamic perforating artery arises from the proximal PCA (P1 segment) between the BA and PCOM and supplies the rostral mesencephalon and both paramedian thalami (9, 10). Proximal embolism is thought to be the most common etiology of stroke in this territory with this variant (9–11).

Asymmetric thalamic involvement is seen in two-thirds of cases and midbrain infarction is present in over half (9, 10). Patients with bilateral paramedian thalamic lesions may develop altered sensorium, vertical gaze palsy, and memory impairment (10). Conventional vascular imaging does not routinely demonstrate these tiny perforating vessels. Hypoplastic or absent P1 segments are more likely to be seen with this variant (11). Other vascular causes of bilateral thalamic injury include venous thrombosis (12), top of the basilar occlusion (13), and hypoxic–ischemic injury (14).

### Incomplete circle of Willis and the fetal PCA

An incomplete COW is present in 48–58% of the population (6) (Figure 2B). A PCA arising directly from the intracranial internal carotid artery (ICA) is defined as fPCA and most are unilateral (Figure 2D). A fPCA is either partial or complete depending on whether or not a hypoplastic P1 segment is present and occurs in 10–29% of the population (6, 15) (Figure 2C). Bilateral fPCAs are associated with a small caliber BA, as the BA does not contribute to mesencephalic, temporal, or occipital lobe flow. There is no established association between unilateral or bilateral fPCAs and stroke risk (15, 16). On the other hand, the etiologic evaluation of occipital stroke in patients with an ipsilateral fPCA should include an assessment for carotid artery disease. Moreover, patients with hemodynamically significant carotid occlusive disease and ipsilateral fPCAs lack the capacity to develop leptomeningeal anastomoses between the anterior cerebral artery (ACA), middle cerebral artery (MCA), and PCA. Patients with ICA occlusion, an ipsilateral fPCA, and a non-functioning ACOM may be particularly vulnerable to ischemia and infarction due to hemodynamic failure compared to those with adequate contralateral flow through a functioning ACOM (17).

### Anatomical variations of the vertebral arteries

Approximately 70% of people have a left dominant VA (4, 5) (Figure 2A). In some, the non-dominant hypoplastic VA congenitally ends in the posterior inferior cerebellar artery (PICA) without joining the BA. VA hypoplasia (VAH) has been associated with PC stroke (5, 18). In a study evaluating the correlation of VA asymmetry and pontine infarctions, patients with VA asymmetry (defined as internal vessel diameter ratio of 1:2 or more) had twice as many pontine infarctions than those with symmetric VAs. The infarcts more commonly occurred ipsilateral to the smaller vessel (18). Another study reporting VAH in 13% of patients with PC strokes found this variant to be more common in PC strokes as compared to matched controls with AC strokes. Moreover, the majority of PC strokes affected the brainstem and cerebellum (5).

Basilar artery curvature may result from congenital asymmetric blood flow to the VB junction causing asymmetric vessel wall tension, displacement, and elongation of the BA (Figure 2A). The resulting inner wall shear stress may cause endothelial injury leading to local thrombosis, torsion of the pontine perforating arteries, or diminished blood flow in the smaller intracranial VA leading to subsequent pre- and post-junction infarctions (4).

**Figure 3:**
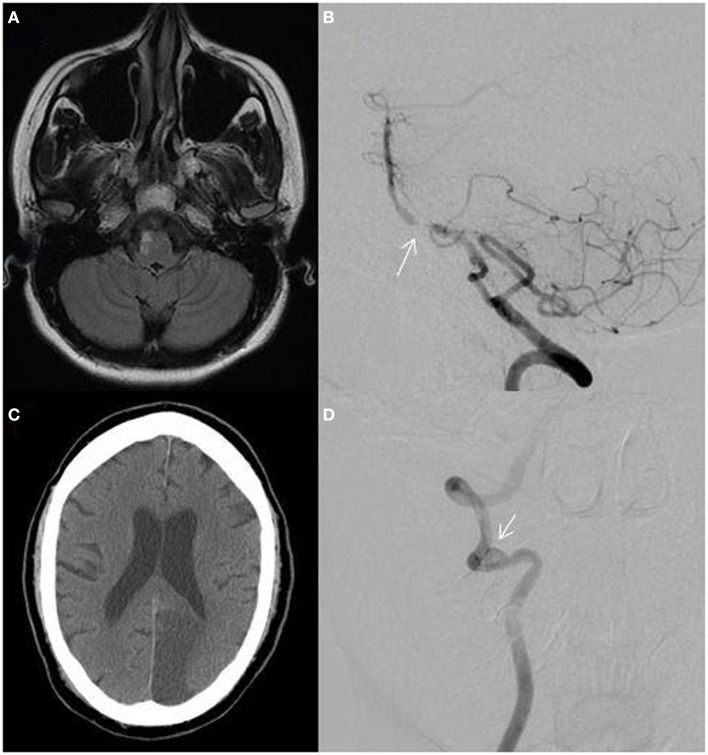
**(A)** MRI fluid-attenuated inversion recovery (FLAIR) sequence showing a right lateral medullary infarction in a 32-year-old woman with a hypoplastic right vertebral artery shown in Figure 2A; **(B)** catheter angiogram of a 55-year-old African-American man showing atherostenosis at the vertebrobasilar junction (arrow); **(C)** non-contrast CT showing a left posterior cerebral artery territory infarction in a 60-year-old man with atrial fibrillation; **(D)** catheter angiogram showing a right distal vertebral dissecting aneurysm with intraluminal thrombus (arrow) in a 19-year-old man presenting with vertigo, ataxia, and a right cerebellar infarction.

The relationship between VAH, BA curvature, and stroke laterality has been evaluated. In one study, 69% of patients with pontine or PICA territory infarctions had VAH and 75% had BA displacement opposite to the dominant VA. Pontine infarction occurred contralateral to the side of the BA displacement in 72% of patients. PICA territory infarction was seen ipsilateral to the VAH in 72% (Figure 3A). In addition, greater difference in VA diameters predicted more severe BA curvature (4).

## Etiologies of Ischemic Posterior Circulation Stroke

Beyond the minority of cases associated with vascular anomalies of the PC previously discussed, several pathological and demographic differences must be considered when determining stroke etiology. Multiple stroke registries have been analyzed to investigate the patient characteristics and most common etiologies of PC stroke.

### Atherosclerosis

Large vessel atherosclerotic disease within the PC can result in thromboembolism, or less commonly, hemodynamic failure leading to ischemia (19). A study of combined registries found large vessel atherosclerotic disease to be responsible for 35% of PC strokes and small vessel disease accounting for 13% (2). The most common locations of VA atherostenosis are the V1 and V4 segment (3, 20). Caucasian men more commonly have extracranial atherosclerosis and evidence of concomitant coronary and peripheral vascular disease (21). Alternate pathology, such as arterial dissection, should be considered when stenosis only involves the V2 and V3 segments. Arterial wall fatty streaks, fibrous plaques, and calcified plaques are less prevalent in the VA as compared to the carotid artery (20).

Steno-occlusive atherosclerotic disease as an embolic source most commonly affects the PICA territory, distal BA, superior cerebellar artery (SCA), and PCA branches (22). AC strokes related to cervical ICA stenosis are associated with thrombogenic plaque characteristics (23). Similarly, lesion irregularity and plaque morphology correlate with severity of ischemic presentation in the PC (24).

The most common intracranial site of atherostenosis is the BA, followed by the ICAs, MCAs, VAs, PCAs, and ACAs. Intracranial stenosis is more prevalent among African-American and Asian individuals and may account for a higher rate of strokes in these populations compared to Caucasians (Figure 3B). Neurovascular imaging techniques such as endovascular ultrasound and high resolution MRI have shown both vulnerable atherosclerotic plaques and fibroproliferative lesions (25).

Ischemia due to intracranial disease may result from tissue hypoperfusion, *in situ* thrombosis, or artery-to-artery thromboembolism. Mild intracranial disease may have minimal effect on cerebral hemodynamics. As the stenosis increases, reflex vasodilation due to inadequate or failing collateral circulation occurs to increase cerebral blood volume (CBV) and preserve normal cerebral blood flow (CBF), and oxygen extraction fraction will increase as CBF further deteriorates. Failure of these compensatory mechanisms is known as “misery perfusion” (26). Patients with tandem extracranial and intracranial lesions or bilateral disease more commonly suffer clinical effects of hemodynamic changes (3, 24). “Tandem lesions” are commonly found in patients with VA atherosclerosis and PC stroke (22).

### Cardioembolism

Approximately 40% of cerebral blood flow goes to each ICA and only 20% goes to the PC. Therefore by chance, a fifth of cardiac emboli may end up within the PC (3). Cardiac diseases with risk for embolism include mechanical prosthetic valves, atrial fibrillation, left atrial or ventricular thrombus, myocardial infarction within 4 weeks of stroke, dilated cardiomyopathy, infective endocarditis, mitral stenosis without atrial fibrillation, bio-prosthetic cardiac valves, and congestive heart failure (Figure 3C).

In 407 patients in the NEMC-PCR, embolism (cardiac, artery-to-artery, or both) was the most common etiology (40%) and a cardiac source of embolism was reported in 24%. Distal PC territory infarctions were most common followed by the middle territory (3). In contrast, the Hallym stroke registry (HSR) reported large artery stenosis in nearly half of 591 Korean patients with PC strokes, intrinsic small vessel disease in a third, and only 11% had potential cardiac sources of embolism. Middle territory infarction occurred in 36.5% of patients followed by the distal territory in 28.1% (27).

### Variations in PC stroke etiology

Similarities and differences in both registries exist. The prevalence of hypertension, diabetes, hyperlipidemia, and smoking were similar in both registries. Over 80% of both cohorts were investigated with magnetic resonance imaging (MRI) and magnetic resonance angiography (MRA). Compared to HSR, the NEMC-PCR included a higher proportion of men (63 vs. 56%) and patients were slightly younger (mean age 61 vs. 63 years). NEMC-PCR patients were predominantly Caucasian (84%) and only a minority was Asian (9.5%) (3).

In the HSR, cardiac risk factors were found less frequently compared to the NEMC-PCR. Transthoracic echocardiography (TTE) was only performed in 37.5%, transesophageal echocardiography (TEE) in 14.1% in contrast to the NEMC-PCR, and VA origins were not routinely evaluated (27). The lower cardioembolic risk of the HSR may have accounted for fewer infarctions in the distal and multiple territories compared to the NEMC-PCR (25, 28). A higher prevalence of intracranial atherosclerotic disease in the Korean population may have accounted for a higher proportion of middle territory infarctions.

### Vertebrobasilar dolichoectasia

Vertebrobasilar dolichoectasia (VBD) refers to dilatation, elongation, and tortuosity of the BA. The anatomy is highly variable. Asymptomatic patients may be found to have VBD incidentally on neuroimaging while other patients present with vertebrobasilar territory ischemia (29). Risk factors for VBD include male gender, increasing age, hypertension, smoking, and history of a myocardial infarction. VBD has been associated with aortic dilations, ectatic coronary arteries, Marfan syndrome, late-onset Pompe disease, autosomal dominant polycystic kidney disease, and Fabry disease (30). The estimated 5-year complications in VBD is 17.6% for ischemic stroke, 10.3% for brainstem compression, 10.1% for TIAs, 4.7% for hemorrhagic stroke, 3.3% for hydrocephalus, and 2.6% for subarachnoid hemorrhage (SAH) (30). Long-term prognosis may be related to VBD severity and evolutionary characteristics such as vertical elongation, lateral displacement, and diametric changes over time (29).

### Arterial dissection

Cervical artery dissections (CADs) may occur spontaneously or result from major or minor cervical trauma (31). In young adults (15–49 years), CADs accounts for approximately 15% of strokes (32, 33). The presence of headache, neck pain, history of trauma, or neck manipulation associated with stroke should raise suspicion (34). VA dissections are most commonly found in the V2 and V3 segments (35). Posterior neck pain is present in half of patients and headache (more commonly occipital) occurs in two-thirds. In addition, VA dissections more frequently present with ischemia (>90%) and PICA territory strokes (lateral medullary and/or cerebellar) are common (36). Isolated neck pain or headache without ischemic symptoms can be observed in 12% (35). Compared to patients with ICA dissections, patients are younger, more commonly have neck pain, are more likely to have associated SAH, and take 2 days longer on average to diagnose (37, 38). Approximately 10% of VA dissections extend intracranial (35). Intracranial extension carries a higher risk of dissecting aneurysm formation, SAH, and mortality (39) (Figure 3D).

### Other etiologies and associations

Other less common causes of ischemia with predilection for the PC circulation include subclavian steal syndrome, giant-cell arteritis, and Fabry disease. Mitochondrial encephalopathy, lactic acidosis, and stroke like episodes (MELAS), migraines, and posterior reversible encephalopathy syndrome (PRES) also have a predilection for the PC (19). Reversible cerebral vasoconstriction syndrome (RCVS) should be considered in the differential diagnosis of sudden onset headache and focal neurological deficits as this may mimic PCA embolus presentation (40).

## Clinical Presentations of Posterior Circulation Stroke

In clinical practice, not all PC stroke presentations are classic. Many patients present with signs and symptoms of multifocal PC infarctions. Moreover, the PC is rich in potential collateral support and clinical manifestations of BA ischemia may be highly variable. Symptoms associated with PC strokes such as diplopia, visual field defects, dysphagia, vertigo, alteration in consciousness, or hearing loss may aid with localization. PC strokes have fewer cortical findings and relatively small lesions can cause significant deficits as compared to AC stroke due to the close proximity of major afferent and efferent tracts and cranial nerve nuclei in the brainstem. Table 1 lists vascular territories of the PC with corresponding clinical findings and classic stroke syndromes.

**Table 1:** **Vascular territories of the PC with corresponding clinical findings**.

Vascular territory	Anatomical location	Stroke syndrome	Clinical findings
**Unilateral PCA**	Occipital lobe	Contralateral homonymous hemianopsia	Homonymous hemianopsia with macular sparing
	
	Dominant occipital lobe *plus* splenium of corpus callosum	Alexia without agraphia	Homonymous hemianopsia and alexia without agraphia
	
	Ventral occipital cortex; infracalcarine	Achromatopsia	Loss of color differentiation contralateral to the side of the lesion, can be associated with a quadrantanopsia
	
	Optic radiation	Inferior quadrantanopsia	Inferior quadrantanopsia
	OR supracalcarine	
	
	Myers loop (temporal lobe) or infracalcarine	Superior quadrantanopsia	Superior quadrantanopsia

Bilateral PCA	Both occipital lobes	Cortical blindness	Bilateral cortical blindness with normal ophthalmological findings
		
		Anton’s syndrome	Cortical blindness with denial of blindness and confabulations or visual hallucinations

**PCA–MCA border zone regions**	Bilateral ventral–mesial occipital–temporal border zones	Prosopagnosia	Inability to recognize familiar faces and/or interpret facial expressions. Retained ability to identify with speech or unique feature (e.g., glasses, facial hair, tattoo, etc.)
	
	Bilateral occipital–parietal border zones	Balint’s syndrome	Optic ataxia (inability to reach targets with visual guidance), oculomotor apraxia (inability to volitionally direct gaze), and simultagnosia (inability to synthesize objects within a visual field)
	
	Unilateral left temporal–parietal border zone	Transcortical sensory aphasia	Impaired comprehension, fluent speech but preserved repetition

PICA	Inferior posterior cerebellar hemisphere, inferior vermis, lateral medulla	Lateral medullary or Wallenberg syndrome Superior cerebellar artery syndrome	Vertigo, nausea, vomiting, ipsilateral facial numbness and dysmetria, Horner’s syndrome, dysphagia, and ataxia dysphonia contralateral hemisensory loss below the face

SCA	Dorsolateral upper brainstem and cerebellum and superior cerebellar peduncle	Superior cerebellar artery syndrome	Ipsilateral limb ataxia, vertigo, nystagmus, dysarthria, and gait ataxia

AICA	Ipsilateral labyrinth, lateral pontine tegmentum, and brachium pontis, ICP	Lateral pontine syndrome	Ipsilateral dysmetria, hearing loss, Horner’s syndrome, choreiform dyskinesia, contralateral thermoanalgesia

Top of the BA	Midbrain, thalamus, and mesial temporal lobes and occipital lobes	Top of the basilar syndrome	Somnolence, peduncular hallucinosis, convergence nystagmus, skew deviation, oscillatory eye movements, Colliers sign (retraction and elevation of eye lids), vertical gaze paralysis

**Mid-BA**	**Lateral and medial pons**	Lateral mid-pontine syndrome	Ipsilateral loss of facial sensation and motor function of the trigeminal nerve, ipsilateral dysmetria
		
		Medial mid-pontine syndrome	Ipsilateral dysmetria, contralateral arm and leg weakness and gaze deviation

Pontine paramedian penetrators	Anteromedial pons	Dorsal mid-pontine syndrome	Ipsilateral nuclear facial palsy, horizontal gaze palsy, and contralateral arm and leg weakness

Short pontine circumferential arteries	Anterolateral pons	Superior medial pontine syndrome	Ipsilateral intranuclear ophthalmoplegia, palatal, facial, pharyngeal and/or ocular myoclonus, dysmetria, and contralateral arm and leg weakness, ocular bobbing

Proximal BA	Lower pons	Locked-in syndrome	Quadriplegia, horizontal gaze paralysis, bifacial, paralysis, and tongue and mandibular weakness. Awareness is spared

VA	Medulla and cervical spinal cord	Medial medullary or Dejerine syndrome (intracranial disease may lead to Wallenberg syndrome)	Contralateral arm and leg weakness, hemibody loss of tactile, vibration, position sense, ipsilateral tongue paralysis

Anterior spinal artery		Anterior spinal artery syndrome	Quadriparesis, bilateral pain and temperature loss, decreased sphincter tone, autonomic instability, and hyperreflexia. Proprioception spared

## Common Symptoms of Posterior Circulation Stroke

Common presenting symptoms of PC stroke include vertigo, imbalance, unilateral limb weakness, slurred speech, double vision, headache, nausea, and vomiting. Exam findings include unilateral limb weakness, gait ataxia, limb ataxia, dysarthria, and nystagmus. Infarcts involving the proximal PC territory may cause dysphagia due to pharyngeal weakness, nausea, vomiting, and Horner’s syndrome. Infarcts involving the middle territory are often associated with limb weakness and nuclear facial palsy. Distal territory infarctions are commonly associated with decreased appendicular sensory loss, lethargy, and visual field defects (41). Patients typically present with more than one finding and rarely have an isolated symptom or sign of PC ischemia (21). The frequency of common presenting signs and symptoms of PC infarcts from the NEMC-PCR (41) and ischemic posterior circulation stroke in state of Qatar registry (IPCSQ) (42) are summarized in Table 2.

**Table 2:** **Frequency of common presenting signs and symptoms of posterior circulation infarcts**.

Symptoms and signs	NEMC-PCR (*n* = 407) (%)	IPCSQ (*n* = 116) (%)
Dizziness or vertigo	47	75
Dysarthria	31	64
Nausea or vomiting	27	60
Loss or alteration of consciousness	5	18
Limb weakness	38	49
Ataxia	31	65
Nystagmus	24	48

*NEMC-PCR, New England Medical Center posterior circulation registry; IPCSQ, ischemic posterior circulation stroke in State of Qatar*.

## Natural History of Posterior Circulation Ischemia

Typical of stroke, symptoms are sudden and maximal at onset. Vertebrobasilar insufficiency (VBI) refers to PC symptomatology due to hemodynamic failure resulting in ischemia. A steno-occlusive lesion at any level due of the VA due to any cause may lead to VBI and a physical examination alone is insufficient for diagnosis (43). A detailed clinical history, evaluation of traditional risk factors, and thorough examination in patients with VB symptomatology is paramount.

**Figure 4:**
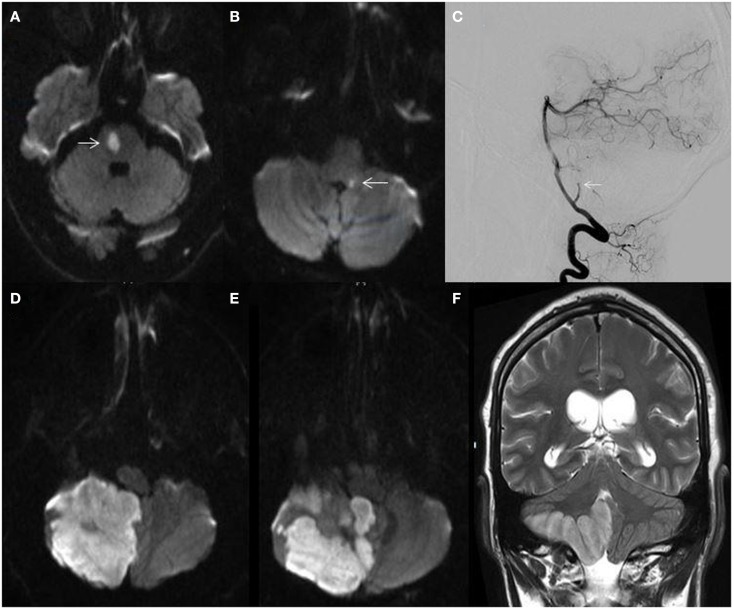
**(A)** MRI diffusion-weighted image (DWI) demonstrating a right ventral pontine infarction (arrow) in a 62-year-old man with fluctuating left sided weakness; **(B)** MRI-DWI showing a small dorsal left medullary infarction (arrow) in a 58-year-old man with hypertension and hyperlipidemia presenting with acute isolated vertigo; **(C)** catheter angiogram showing cut-off of the right posterior inferior cerebellar artery (arrow); **(D,E)** MRI-DWI showing massive right cerebellar hemispheric and vermian infarction; **(F)** MRI T2-weighted sequence demonstrating right cerebellar infarction with edema and mass effect.

Vertebrobasilar TIA may precede PC stroke in approximately one-fourth of cases (3). Episodes of diplopia, vertically oriented binocular visual field loss, vertigo, ataxia, impaired sensorium, or “crossed-findings” (ipsilateral cranial nerve deficits and contralateral long tract signs) are localizing. History of neck pain, trauma, or headache particularly in younger patients may suggest vertebral dissection. Fleeting or stuttering vertebrobasilar symptoms are concerning and should prompt evaluation of basilar artery patency. Distal and mid-basilar artery occlusions typically result in abrupt events without prodromal signs or symptoms as compared to proximal lesions where a fluctuating and progressive course is observed (44). Fluctuating symptoms in the setting of a patent basilar artery was described by Saposnik and colleagues as the “pontine warning syndrome” and characterized by episodes of motor or sensory dysfunction, dysarthria, or ophthalmoplegia due to basilar branch artery disease leading to ventral pontine infarctions (45) (Figure 4A).

History of unilateral arm pain with exertion associated with VBI symptomatology may suggest proximal subclavian artery stenosis causing reversal of normal flow within the ipsilateral vertebral and basilar arteries, known as the subclavian steal syndrome (46). A blood-pressure differential (BPD) of >20 mm Hg between both arm measurements aids in identifying patients with subclavian stenosis (47), however two-thirds of patients with subclavian steal may be asymptomatic. Symptomatic patients commonly have concomitant carotid disease which may provoke VB symptoms (46). Patients with a higher BPD measurement (>40–50 mm Hg) and complete steal phenomenon would more likely benefit from intervention (47).

Vertebrobasilar symptoms or syncope with head-turning known as rotational vertebrobasilar insufficiency or bow hunter’s syndrome is rare. This is most commonly caused by compression of the vertebral artery due to cervical bony osteophyte in older patients, C1–C2 hypermobility, or even neck muscle hypertrophy in younger patients (48, 49).

## Non-Specific Symptoms of Posterior Circulation Ischemia

### Dizziness and vertigo

Dizziness, albeit descriptive, is a non-specific term that is commonly used interchangeably with vertigo by patients. Moreover, patients commonly use dizziness to describe feeling faint or light-headed. Nearly 7.5 million patient visits per year in the ambulatory setting are due to dizziness as the presenting symptom (50, 51). In a study of 1666 patients presenting to the emergency department with complaints of dizziness, vertigo, or imbalance; stroke or TIA was diagnosed in only 3.2% (33 ischemic strokes, 17 TIAs, 1 ICH, and 2 undetermined) (51). Patients with cerebrovascular events were older and more likely to have two or more stroke risk factors than those with other etiologies of their symptoms.

In contrast, in 240 patients clinically diagnosed with vestibular neuritis due to vertigo lasting >24 h without accompanying neurological symptoms and a normal neurologic examination, cerebellar infarctions were found in 10% of patients. Almost all infarctions were within the PICA territory (52). However, only 17% of patients with PICA territory strokes presented with signs and symptoms consistent with vestibular neuritis. The investigators termed this phenomenon, “pseudo-vestibular neuritis.” These findings underscore the non-specific nature of these symptoms, need for careful clinical assessment, and importance for appropriate imaging consideration (Figure 4B).

Qualitative descriptions may be less dependable than estimates of the duration and situations that preceded or provoked the event (53). Brief, vertiginous episodes that occur frequently and begin soon after head movement are more consistent with benign positional vertigo than ischemia (50, 53). Symptoms that increase in frequency or severity should raise more concern (54). Central vertigo may or may not be exacerbated by head positioning. When central vertigo occurs after head positioning, there is usually no latency unlike the latency seen with benign paroxysmal positional vertigo as an otoconial particle travels within the endolymph of the semicircular canals.

Ischemia associated with vertigo is often accompanied by other brainstem or cerebellar signs and symptoms (53). Provocative maneuvers such as the head thrust and Dix–Hallpike maneuvers can help differentiate between central and peripheral etiologies. In the Dix–Hallpike maneuver, patients whose symptoms are reproduced in the supine position with the head tilted down and rotated after 5–10 s latency with or without observable rotational nystagmus are likely to have a peripheral etiology (50). Symptoms and nystagmus that occur immediately upon positioning may occur with both peripheral and central etiologies. Patients that exhibit a corrective saccade after a head thrust in the direction of the dysfunctional side with eyes fixed invariably have peripheral vertigo (52, 54). In contrast, none of the patients with “pseudo-vestibular neuritis” due to a PICA territory infarction in the aforementioned study had a positive head thrust test. Peripheral vertigo may be associated with tinnitus or hearing loss (50) and typically does not present with pupillary abnormalities, dysconjugate gaze, dysmetria, motor weakness, or depressed level of consciousness. The presence of any of these suggests a central etiology. Vertical nystagmus and direction-changing nystagmus are also specific for central lesions.

In patients presenting with acute vestibular symptoms (vertigo, nausea, vomiting, head motion intolerance, and/or nystagmus), the HINTS method, a three-step bedside evaluation composed of horizontal head impulse testing, nystagmus evaluation, and testing of skew can be easily performed in about 1 min. A negative (normal) head impulse, alternating nystagmus, and presence of skew (refixation on cover–uncover testing) has a sensitivity and specificity of 100 and 96%, respectively, for detecting stroke. In addition, the presence of skew deviation is highly specific for brain stem dysfunction in patients with the aforementioned symptoms (55).

### Ataxia

Gait ataxia may be central, vestibular, or sensory in origin. It is almost always present in PC stroke involving the brainstem and cerebellum (56) and nearly all patients with cerebellar ataxia fall toward the lesion side (57). When stratified by infarction site, the frequency of gait ataxia is similar across all territories but tends to be most severe when the cerebellum and cerebellar tracts of the brainstem are involved (56). Furthermore, ataxia associated with a central lesion tends to begin abruptly with full severity at onset (58). Lack of gait evaluation and coordination testing in patients with ataxic symptoms is a common cause of misdiagnosis of cerebellar infarction (59). Dysmetria assessment with finger–nose–finger or heel-to-shin testing and clumsiness with rapid alternating movements (i.e., dysdiadochokinesia) may help evaluate cerebellar dysfunction (50).

### Headache

Headaches occur commonly in the general population due to myriad etiologies and are non-specific symptoms in many cases. The frequency of headache with stroke is approximately 27%, and increases to 46–70% when the PC is involved (60). Headaches associated with PC territory infarcts may be due to irritation of trigeminovascular afferents densely located in brainstem arteries (60). Patients with cerebellar infarcts and unilateral headaches typically have lesions in the ipsilateral cerebellum (57). Table 3 summarizes differentiating features of peripheral and central originated vertigo, nystagmus, ataxia, headache, as well as test finding differences.

**Table 3:** **Differentiating features of peripheral and central originated vertigo, nystagmus, ataxia, and headache**.

Summary of peripheral vs. central etiology distinguishing features		
	Peripheral cause	Central cause
**VERTIGO**		
Onset	Acute or gradual	Acute
Duration	Minutes to hours	Days to weeks
Impact of head movement	Worsens	Variable
Auditory symptoms	Frequent	Often absent
Dix–Hallpike	Positive	Negative
Associated neurological findings	Absent	Often present
**NYSTAGMUS**		
Direction of fast-phase	Unidirectional	Can be alternating
Vertical component	Absent	Can be present
Fatigability	Fatigable in 30–60 s	Not fatigable
Presence of vertigo symptoms	Always present	Can be absent
**ATAXIA**		
Gait ataxia	Present but less severe	Very severe
Truncal ataxia	Uncommon	Common
Cerebellar testing	Normal	Frequently abnormal
Onset	Acute or gradual	Acute
Severity at onset	Less likely to be severe at onset	More likely to be maximal at onset
Headache	Uncommon	Common
Location	Variable	Occipital
Unilateral	Variable	Commonly unilateral
Onset timing	Variable	Typically at time of other symptoms
**HINTS**		
Head impulse test	Abnormal (gaze correction)	Normal
Nystagmus	Fast-phase in one direction	Fast-phase alternating directions
Test of skew	Skew absent	Skew present

## Neuroimaging in PC Stroke

Brain computed tomography (CT) is typically performed as the initial imaging modality for patients presenting with acute stroke symptoms. Unfortunately, CT provides suboptimal visualization of the posterior fossa structures due to obscuration by artifacts produced by the bony structures of the cranial base and early ischemic changes may not be visible. In contrast, MRI provides better visualization of the soft tissue structures (61) and is superior for detecting early evidence of infarction with diffusion-weighted imaging (DWI) sequences. However, in the acute setting, MRI is more time consuming and in patients with metallic foreign bodies, incompatible pacemakers, or claustrophobia it cannot be performed safely.

Subtle hypodensities, loss of gray–white matter differentiation and sulcal effacement have been used to asses for signs of early ischemia on non-contrast head CT. The Alberta Stroke Program Early CT Score (ASPECTS) provides a reliable and standardized topographic assessment of acute ischemic changes in the MCA territory with predictive value (62, 63). In contrast, early ischemic signs on CT in the PC are not as well-established (64). This may be in part due to the smaller area interpreted and density of the posterior fossa. The Posterior Circulation Acute Stroke Prognosis Early CT Score (pc-ASPECTS) has been proposed (65). This score is more sensitive for detection of early ischemic change and prediction of functional outcomes with contrast infusion (as compared to non-contrast CT), and could help identify patients with BA occlusion who are unlikely to have favorable outcomes despite recanalization (65). Infarction size in the PC does not correlate well with stroke severity (66). Due to the close proximity of vital tracts and nuclei, location site is a more critical functional outcome predictor (67). Applying the pc-ASPECTS score to MRI-DWI has been shown to be a powerful marker for the prediction of functional outcome of PC stroke (68).

Despite the superior sensitivity of MRI-DWI in detection of PC stroke compared to CT, MRI can rarely be negative for small infarctions (19). In one study, DWI imaging was falsely negative in 5.8% of all patients with stroke symptoms lasting >24 h when imaging was obtained within 48 h of onset. The false-negative DWI rate for PC stroke was almost 10 times higher than that of the AC (19 vs. 2%). Moreover, up to a third of patients in that study presenting with vertebrobasilar ischemic symptoms who had an initial false-negative DWI study during the first 24 h had positive studies on follow-up (69). Similarly, a 12% false-negative DWI rate was reported for patients with acute vestibular symptoms and PC stroke, when imaging was done within 48 h of symptom onset (55). These studies highlight the importance of adequate history taking, thorough neurologic examination, and a high index of suspicion during patient evaluation beyond sole reliance on neuroimaging.

Presence of a hyperdense BA sign in the setting of acute PC stroke may be indicative of thrombosis. This finding has been shown to be 71% sensitive, 98% specific, and 94% accurate for BA occlusion with a positive predictive value of 83%, negative predictive value of 95%, and a strong predictor of short- and long-term outcome (70). The gold standard for intracranial and extracranial vascular imaging remains the conventional catheter cerebral angiogram (Figure 4C). CT angiography (CTA) and MRA are quick non-invasive imaging modalities with good sensitivity and specificity for large vessel abnormalities. Contrast-enhanced MRA has a higher sensitivity and specificity for detection of vertebrobasilar stenosis >50% as compared to CTA or ultrasound (71). Cervical duplex ultrasound has limited ability to visualize the V1 segment and does not visualize the V4 segment. Additionally, the density of the vertebrae precludes insonation of the portions of the V2 segment within the transverse foramina. Insonation with transcranial Doppler ultrasound through the foramen magnum is able to detect velocity changes in the V4 segment consistent with stenosis but does not routinely obtain gray scale images of the vessel wall to define the cause of stenosis and is operator-dependent. Extracranial VA dissections may be identified by Doppler color-flow studies. Typical findings include irregular stenosis or localized increase in vessel diameter, dissecting membrane with true and false lumen, intramural hematoma, and tapering stenosis with distal occlusion (72). Use of contrast-enhanced MRA increases the sensitivity for detection of dissections (73). Although MRA has good sensitivity for vertebral dissection, it has low specificity (74) unless combined with cervical axial T1 fat saturation sequences which may detect crescent-shaped high intensity signal within the pseudo-lumen suggesting intramural hematoma. CTA is superior to MRA for identifying intimal flaps, pseudoaneurysms, and high-grade stenosis than MRI (75).

## Management

### Management of vertebrobasilar atherosclerotic disease

Ideal treatment of symptomatic vertebrobasilar stenosis and indications for invasive treatments remains a topic of debate as randomized controlled trials data are lacking. Patients with symptomatic vertebrobasilar stenosis, defined as VA or BA stenosis >50% by CTA or contrast-enhanced MRA have a threefold higher 90-day risk of stroke or TIA after a first event as compared to patients without. Stroke risk is highest within the first 2 weeks and patients with intracranial stenosis harbor an even higher risk of early recurrence, up to 33% as compared to 16.2% for extracranial stenosis (1). Management includes treatment of vascular risk factors, statins, and antiplatelet agents similar to AC disease. Earlier reports of a supraclavicular approach for endarterectomy (76) have not been tested in a controlled manner. Bypass procedures are technically challenging and carry a combined morbidity and mortality of up to 17% (77, 78). Current indications for surgical intervention are few such as bypass-grafting or stenting for subclavian steal (79) or surgical decompression for an externally compressed vertebral artery (80). Some practitioners have adopted the combined use of aspirin with a 90-day course of clopidogrel after minor stroke or high-risk TIA patients based on the low stroke rate and similar bleeding profile in the stenting and aggressive medical management for preventing recurrent stroke in intracranial stenosis (SAMMPRIS) trial (81). Additionally, a study of a Chinese population compared aspirin monotherapy to clopidogrel with aspirin for 90 days in patients with acute minor stroke or TIA. This study demonstrated safety and a modest benefit of combination therapy over aspirin (82).

In the Carotid and Vertebral Artery Transluminal Angioplasty Study (CAVATAS), only eight patients underwent endovascular angioplasty with or without stenting precluding meaningful conclusion about the utility of endovascular intervention within the VA (83). A recent systematic review on stenting of the extracranial vertebral artery showed a 1.1% 30-day peri-procedural stroke rate, and 2-year in-stent restenosis rate of 11–30% for drug-eluting stents and bare metal stents, respectively (84). A non-randomized prospective single center study evaluating 114 patients from the Borgess Medical Center vertebral artery ostium registry who underwent ostial stenting for symptomatic vertebral stenosis of >50% showed a 2% stroke recurrence rate at 1 year and a 25% restenosis rate >50% irrespective of stent type used (bare metal or drug-eluting) (24).

In the stenting of symptomatic atherosclerotic lesions in the vertebral or intracranial arteries (SSYLVIA) trial, the 30-day perioperative stroke rate for intracranial stenting was 6.6% (85). Restenosis at 6 months was reported in 35% of patients and nearly a third were symptomatic. Intracranial angioplasty with stenting using the Wingspan stent plus aggressive medical management (AMM) was compared to AMM alone in the SAMMPRIS trial (81). AMM included antithrombotic therapy with aspirin plus clopidogrel for 90 days, statins for target LDL ≤70 mg/dL, blood-pressure lowering according to the seventh report of the Joint National Committee on treatment of prevention, detection, evaluation, and treatment of high blood pressure, and lifestyle changes to achieve better glycemic control, weight loss, and regular exercise. Patients randomized to angioplasty with stenting had nearly three times the rate of stroke and death at 30 days compared to the medical arm and the trial was prematurely stopped due to the high complication rate in the procedure arm. There were no differences in stroke distal to the target lesion beyond 30 days up to a median of 32.4 months between the treatment groups (86). Not only did the angioplasty and stenting arm exceed the anticipated 30-day stroke and death rate, the AMM only arm had fewer events than expected (81, 86). The reasons for better-than-expected outcomes in the latter are to date uncertain. However, the rapid reduction in low-density lipoprotein cholesterol and blood pressure and the potential benefits of short-term dual antiplatelet agent use may have contributed. Only 13% of patients in SAMMPRIS had vertebrobasilar stenosis. However, of the strokes seen within 30 days in the angioplasty and stenting arm, most occurred at the time of the procedure and the majority of those were due to BA paramedian branch occlusions (87).

### Management of vertebrobasilar dolichoectasia

Specific guidelines for treatment of VBD are lacking. Management is typically conservative with use of antiplatelet agents or anticoagulants. However, antithrombotic use may be associated with an increased risk of intracerebral hemorrhage (88). Ventriculoperitoneal shunting for hydrocephalus, microsurgery for cranial nerve compressions, and superficial temporal artery–superior cerebellar artery bypass have been reported (30).

### Management of cardioembolic PC stroke

Patients with PC stroke and atrial fibrillation should be managed with long-term anticoagulation. Available oral agents include vitamin K antagonist therapy with warfarin (target INR 2.5; range 2–3), direct thrombin inhibitors such as dabigatran, and factor Xa inhibitors such as apixaban, edoxaban, and rivaroxaban (89). Patients with metallic prosthetic cardiac valves should receive warfarin. Optimal antithrombotic treatment for patients with other cardioembolic predisposing factors is at present uncertain and needs to be individually tailored. A detailed discussion of this topic is beyond the scope of this review.

### Management of acute PC stroke

Acute treatment options for PC stroke include IV recombinant tissue-plasminogen activator (IV-rt-PA), intra-arterial fibrinolysis, and endovascular thrombectomy. The acute management of ischemic stroke is primarily governed by time from last known well and comorbid conditions. Within the therapeutic treatment window, stroke severity based on the NIHSS play an important role in decision-making. However, this measure has limitations in PC stroke. Higher points in AC stroke are seen with cortical findings and motor deficits, in contrast to fewer points assigned to cranial nerve deficits and ataxia which may occur without weakness in PC strokes (64, 90). Patients with PC strokes may have an unfavorable 3-month outcome despite relatively low NIHSS scores (90).

### Intravenous thrombolysis

Only 5% of the National Institute of Neurological Disorders and Stroke (NINDS) Study that led to U.S. Food and Drug Administration approval of alteplase for acute ischemic stroke had PC stroke (91) and data regarding PC strokes in the European Cooperative Acute Stroke Study III (ECASIII) was lacking. Clinical practice guidelines in the U.S and Europe recommend IV-tPA as first-line therapy for patients with ischemic stroke within 4.5 h of last known well (92). The Third International Stroke Trial (IST 3) showed that treatment with IV-tPA within 6 h of symptom onset was associated with improvements in functional outcome and quality of life without effect on overall survival rate (93). However, only 246 of 3025 (8.1%) randomized patients had PC strokes. Studies evaluating the effectiveness of other IV fibrinolytics including tenecteplase and desmoteplase with potentially longer treatment windows are ongoing (94, 95).

Basilar artery occlusion (BAO) is a neurological emergency that carries >80% fatality rate without treatment (96, 97) prompting heroic efforts beyond the IV thrombolytic time constraints. BAO has variable presentation depending on the collateral flow and individual ischemic tolerance. A study of patients treated with IV-thrombolysis followed by full dose heparin showed that recanalization of BAO up to 48 h from symptom onset produced good outcomes in 50% of patients independent of time to treatment (98). A Japanese study reported the outcomes of 25 patients with BAO treated with low dose IV-rt-PA (0.6 mg/kg). Recanalization during hospitalization occurred in 78% of patients and 44% of patients were independent at 3 months. These patients had lower NIHSS scores at baseline, shorter onset-to-treatment times, and greater frequency of thromboembolic stroke etiology which may have accounted for the more favorable results compared to other reports (99).

### Intra-arterial fibrinolysis

The use of intra-arterial (IA) thrombolytics in PC stroke has been previously studied (96, 97, 100, 101). Favorable outcomes including improved survival after IA-thrombolysis for patients with stroke due to BA occlusion has been associated with angiographic evidence of recanalization (100). The recanalization rate of occlusions in the vertebrobasilar system from IA therapy alone is approximately 63% (102). An analysis of 420 non-randomized patients with BAO treated with IA-thrombolysis (82%) or IV-rt-PA (18%) showed IA-thrombolysis more frequently achieved recanalization than IV-rt-PA (65 vs. 53%). However, death or dependency and favorable functional outcomes were not significantly different. Regardless of treatment modality, a good outcome was achieved in only 2% of those without recanalization, underscoring the devastating nature of BAO and importance of BA recanalization (96). Establishing the ideal treatment modality for BAO has been challenging. An early randomized controlled trial of IA thrombolytic infusion in patients with BA occlusion was too small to demonstrate meaningful conclusions (97). The Basilar Artery International Cooperation Study (BASICS) prospectively observed and compared outcomes of patients with BAO and found no conclusive evidence of superiority for IA-thrombolysis over IV-thrombolysis and prompted the need for a randomized control trial (103). The American Heart Association/American Stroke Association recommends IA-rt-PA in select patients with MCA occlusion within 6 h of symptom onset but no recommendation is made for IA-thrombolysis in PC stroke including BA occlusion (104).

### Mechanical thrombectomy

Recanalization has a strong association with clinical outcome. A large meta-analysis of 53 studies with reported recanalization rates including 2066 patients demonstrated a four- to fivefold increase in the odds of a good functional outcome and similar decrease in the odds of death in patients with successful recanalization (102). In addition, patients with partial or complete early recanalization fare better than those with delayed recanalization or persistent occlusions (105–107). Mechanical thrombectomy achieves high recanalization rates for all target vessels in reported device trials. Recanalization is more readily achieved with the stent retrievers than older generation devices such as coil retrievers and aspiration catheters (108) though data on patients with PC stroke is limited. Only 10% of strokes included in the Merci trial were in the PC with 50% success achieving partial or complete recanalization (105). In the Multi-Merci trial, 8.5% of patients had PC stroke with an 86% successful recanalization rate (109) and 9% in the Penumbra Pivotal study with no specific data regarding PC recanalization (107).

Recent randomized controlled trials have not clarified the role of endovascular therapy for acute ischemic stroke in general and PC stroke in particular. The interventional management of stroke III (IMS III) trial, showed no overall benefit of endovascular rescue therapy after IV-rt-PA compared to IV-rt-PA alone in 656 randomized patients with ischemic stroke and NIHSS ≥10. However, older generation thrombectomy devices were employed, onset-to-treatment times were long, and only 2% of patients had PC stroke. A favorable trend was seen for patients receiving IV-rt-PA within 2 h of symptom and endovascular rescue therapy within 90 min of IV-rt-PA initiation (110). The SYNTHESIS expansion trial randomized patients with ischemic stroke to IV-rt-PA or IA-thrombolysis with optional mechanical thrombectomy. Overall, endovascular treatment was not found to be superior to IV-tPA alone, few patients received mechanical thrombectomy and only 8% of randomized subjects had PC stroke (111). PC strokes were systematically excluded from the magnetic resonance and recanalization of stroke clots using embolectomy (MR RESCUE) trial evaluated endovascular therapy which concluded that a favorable penumbral pattern on neuroimaging was not able to identify patients who would differentially benefit from endovascular therapy for acute ischemic stroke in the AC within 8 h (112). Multiple critiques following these publications have raised issues with study design, infrequent use of newer devices, patient selection, and dosing of IV thrombolytic among others. Despite these criticisms and the fact that PC strokes were under-represented, the conclusions from these studies have thus far been discouraging (113).

### Management of vertebrobasilar dissections

The main mechanism of stroke after cervical arterial dissection is thromboembolism rather than hemodynamic compromise. This prompts consideration regarding antithrombotic selection for recurrent stroke prevention (36). Antithrombotic agents including anticoagulants or antiplatelet agents are typically employed. The use of either therapy may depend on stroke severity, infarct size, bleeding risk, the presence of free floating thrombus, or intracranial extension of the dissection. When anticoagulation is chosen, a short period of 3–6 months is usually employed (114). The majority of dissection heal within 2–3 months with >90% resolution of stenosis (36). Typically, intracranial extension of a dissection carries a higher risk of subarachnoid hemorrhage and anticoagulation is not recommended. The only randomized controlled trial evaluating the effectiveness of antiplatelet therapy compared to anticoagulation for patients with acute cervical arterial dissections is currently underway (115). In patients with acute occlusion or dissecting aneurysms, stent-assisted coil embolization or double stent placements may be an alternative treatment (116).

### Post stroke considerations for the posterior fossa

Cerebellar infarctions can rapidly swell due to cytotoxic edema within the tight constraints of the non-yielding posterior fossa and lead to obstructive hydrocephalus or fatal herniation if not adequately managed in a timely fashion (117–119) (Figures 4D–F). Patients should be clinically monitored for signs of increased intracranial pressure such as headache, vomiting, lethargy, disorientation, or neurologic deterioration, as well as evidence of hypertension, bradycardia, or irregular respiratory pattern. Acute temporizing measures would include hyperventilation, head elevation to 30°, osmotherapy, blood-pressure management to maintain cerebral perfusion, and sedation until definitive surgical treatment is started (120). Patients who receive surgical treatment fare better than those only medically managed, with better functional outcomes and lower mortality (119). Unilateral cerebellar infarctions may be managed with ventriculostomy, suboccipital decompression with or without removal of necrotic tissue, or a combination of all three. In a study of patients with unilateral cerebellar infarctions with a median Glasgow Coma Scale (GCS) of 9 that received all three surgical treatments, GCS scores improved to 13.6 at discharge and mortality was 31% (118). In patients with malignant cerebellar edema, bilateral suboccipital decompressive craniectomy has been shown to be safe with a 6-month mortality of 28% (117).

## Conclusion

Several anatomical and clinical differences distinguish PC infarctions from those in the AC and have practical implications. Vascular anatomical variation may affect stroke severity and presenting signs and symptoms. Large vessel atherostenosis is common but cardioembolic strokes, dissections, and other etiologies are known to affect the PC. Quantitative measures such as the NIHSS alone may not suffice and clinical signs and symptoms such as dizziness, vertigo, and ataxia may be diagnostic pitfalls. MRI is superior to CT for imaging an acute PC infarct but may rarely miss a small infarct early on. A variety of non-invasive vascular imaging modalities are available to aid the etiologic work-up and have replaced conventional catheter angiography in many circumstances. IV-rt-PA is the recommended first-line treatment for eligible patients. The role of endovascular acute revascularization therapy remains uncertain at present. The cornerstone of recurrent stroke prevention includes risk factor control primarily blood-pressure lowering, lipid-lowering with statins, and antithrombotic therapy. Aggressive medical management has been shown to be superior to endovascular angioplasty and stenting in patients with a range of intracranial atherostenosis including lesions in the PC.

## Conflict of Interest Statement

The authors declare that the research was conducted in the absence of any commercial or financial relationships that could be construed as a potential conflict of interest.
